# Mitochondrial haplotype diversity in the tortoise species *Testudo graeca *from North Africa and the Middle East

**DOI:** 10.1186/1471-2148-5-29

**Published:** 2005-04-18

**Authors:** Antoinette C van der Kuyl, Donato LP Ballasina, Fokla Zorgdrager

**Affiliations:** 1 Dept. of Human Retrovirology, Academic Medical Center, University of Amsterdam, Meibergdreef 15, 1105 AZ Amsterdam, The Netherlands; 2 Centro CARAPAX, CP34, 58024 Massa Marittima (Gr), Italy

## Abstract

**Background:**

To help conservation programs of the endangered spur-thighed tortoise and to gain better insight into its systematics, genetic variation and evolution in the tortoise species *Testudo graeca *(Testudines: Testudinidae) was investigated by sequence analysis of a 394-nucleotide fragment of the mitochondrial 12S rRNA gene for 158 tortoise specimens belonging to the subspecies *Testudo graeca graeca*, *Testudo graeca ibera*, *Testudo graeca terrestris*, and a newly recognized subspecies *Testudo graeca whitei*. A 411-nucleotide fragment of the mitochondrial D-loop was additionally sequenced for a subset of 22 *T. graeca*, chosen because of their 12S gene haplotype and/or geographical origin.

**Results:**

Haplotype networks generated by maximum-likelihood and neighbor-joining analyses of both the separate and the combined sequence data sets suggested the existence of two main clades of *Testudo graeca*, comprising *Testudo graeca *from northern Africa and *Testudo graeca *from the Turkey and the Middle East, respectively.

**Conclusion:**

Mitochondrial DNA haplotyping suggests that the tortoise subspecies of *T. g. graeca *and *T. g. ibera *are genetically distinct, with a calculated divergence time in the early or middle Pleistocene. Other proposed subspecies could not clearly be recognized based upon their mt haplotypes and phylogenetic position, and were either part of the *T. g. graeca *or of the *T. g. ibera *clade, suggesting that genetic evidence for the existence of most of the 15 proposed subspecies of *T. graeca *is weak.

## Background

*Testudo graeca *Linnaeus 1758, or the spur-thighed tortoise, is an endangered species with a broad distribution range. It can be found in northern Africa (e.g. Morocco, Algeria, Tunisia and Libya), the Middle East (e.g. Israel, Lebanon, Jordan, Syria, and Iraq), Europe (Bulgaria, Romania, Turkey, Greece, and multiple introductions into Spain and Greece), and in Asia (e.g. Armenia, Azerbaijan, Georgia, Turkmenistan, Iran, and possibly Afghanistan). Surprisingly, *T. graeca *is absent from Egypt [1]. Speciation and subspeciation in the genus *Testudo *are still highly debated, and are notoriously complicated in *Testudo graeca*. Four subspecies have been recognized based upon morphology [2] and are generally accepted, but many more have (often unofficially) been postulated ([3,4] and several others). The latest revision of *T. graeca *systematics now includes 15 subspecies [5]: *T. g. graeca *Linnaeus 1758, *T. g. anamurensis *Weissinger 1987, *T. g. antakyensis *Perälä 1996, *T. g. armeniaca *Chkhikvadze & Bakradze 1991, *T. g. buxtoni *Boulanger 1920, *T. g. cyrenaica *Pieh & Perälä 2002, *T. g. floweri *Bodenheimer 1935, *T. g. ibera *Pallas 1814, *T. g. nabeulensis *Highfield 1990, *T. g. nikolskii *Chkhikvadze &Bakradze 2002, *T. g. perses *Perälä 2002, *T. g. soussensis *Pieh 2001, *T. g. terrestris *Forskål 1775, and *T. g. zarudnyi *Nikolsky 1896. An overview of *Testudo graeca *taxonomy is also available from the EMBL reptile database [6]. However, many novel descriptions have not been extended with population research or DNA analysis, and thus the contribution to the classification of *T. graeca *remains unclear. Two dwarf forms of *T. graeca *that developed in North Africa have been described as a new species or even another genus: *Testudo flavominimaralis *and *Furculachelys nabeulensis *[3]. A rather large, highly domed form, originally named *Testudo whitei *Bennett, 1836, was moved into a new genus: *Furculachelys whitei *[7]. This latter classification could not be confirmed by mitochondrial DNA analysis [8].

Of the generally accepted subspecies, *T. g. graeca *of northern Africa, *T. g. ibera *from the Republic of Georgia, Bulgaria, North-eastern Greece, Turkey (with the exception of the Black Sea coast), Iran, and northern Iraq, and *T. g. terrestris *from southern Turkey, Syria, Lebanon, Jordan and Israel, are the best described. The fourth subspecies, *T. g. zarudnyi*, is restricted to central and eastern Iran and Afghanistan, while *T. g. nikolskii *was reported to inhabit dry subtropic ecosystems along the north eastern Black Sea coast [9]. *T. g. graeca *has been introduced in recent times in southern Spain and on the Spanish island of Mallorca (see [10]). A morphological study suggested that the first three subspecies should be elevated to full species level [11]. Furthermore, it was recently hypothesized based on genetic analysis that *T. graeca *of western Morocco constitutes a separate subspecies [8,10], provisionally named *Testudo graeca whitei*. Morphologically it was assigned full species status in an earlier study [7]. *T. graeca *shows variable colouring and patterning, which are greatly influenced by environmental factors and make subspecies identification difficult [12], especially in captive animals of unknown provenance. DNA analysis could also be helpful in subspecies determination [13].

*T. graeca *numbers are steadily declining in the wild, partly due to agricultural developments and partly because the species is one of the most popular tortoises on the pet market, although its protected status and associated regulations have lessened much of the trade. For example export from Morocco has been illegal since 1978, and import into the European Community has been forbidden since 1984. Because the systematics of the species complex is unclear, and the genetics of wild *T. graeca *populations has not been investigated, conservation and reintroduction programs are difficult to establish. *T. graeca *can be bred in captivity, and captive offspring could thus be used to replace wild populations (or be used for the pet market to protect wild animals from illegal trade).

Using samples obtained from 158 *Testudo graeca*, fragments of the mitochondrial (mt) 12S rRNA gene and D-loop sequence were analysed to estimate haplotype diversity and genetic distances in this tortoise complex. The 12S rRNA gene has previously been used to examine genetic variation in *T. graeca *from Morocco and from Spain [10]. In this study, three 12S rRNA gene haplotypes were found in 19 tortoises from six locations in these countries. The mt 12S rRNA gene was also used to elucidate phylogenetic relationships in the genus *Testudo *[8]. In the latter study a total of ten 12S haplotypes were detected in 28 *T. graeca *specimens originating from 9 locations in Africa and Asia.

## Results and discussion

### Mitochondrial haplotype variation in *Testudo graeca*

The 12S rRNA fragment amplified was 393–394 nucleotides in length in our *Testudo graeca *data set, and 10 positions were variable (including a single nt deletion in one individual). A total of fourteen mt 12S haplotypes were observed in 158 unrelated individuals (Table 1), and six haplotypes were seen only once (Table 2). Five of these haplotypes differed by a single nt change from more frequent haplotypes; one had a single nt deletion. The single nt differences cannot easily be attributed to PCR artifacts, as the analysis was done by direct sequencing of PCR fragments. The D-loop fragment amplified was 410–411 nucleotides in length in the twenty-two selected *T. graeca*, and 20 positions were variable (including a single nt insertion in one individual). A total of 13 mt D-loop haplotypes were observed in 22 *T. graeca *(Table 3), of which 6 were seen only once. Four of these differed by a single nt change from other, more frequent haplotypes, while one had a single nt insertion, and one differed by 5 nt from the nearest haplotype. The combined data set contained 16 different mt haplotypes.

**Table 1:** Nucleotide variation in a 12S rRNA gene fragment of captive and wild-caught *Testudo graeca*

**15**	**27**	**85**	**183**	**194**	**216**	**219**	**224**	**244**	**253**	**326**	**328**	**339**	**Haplotype**
C	T	A	A	G	A	T	C	C	T	G	T	G	**Tg1.0**
C	T	A	A	G	A	T	C	C	T	G	T	A	**Tg1.1**
C	T	A	C	G	A	T	C	C	T	G	T	G	**Tg2.0**
C	T	A	C	G	A	T	C	C	T	G	C	G	**Tg2.1**
C	T	A	C	G	A	T	T	C	T	G	T	G	**Tg3.0**
C	T	A	C	G	A	T	T	C	-	G	T	G	**Tg3.1**
C	C	A	C	A	G	T	C	C	T	G	T	G	**Tg4.1**
C	C	A	C	G	G	T	C	C	T	G	T	G	**Tg4.2**
C	C	A	C	G	A	T	C	T	T	G	T	G	**Tg4.3**
C	C	A	C	G	A	T	C	C	T	G	T	G	**Tg5.0**
T	C	G	C	G	A	C	C	C	T	G	T	G	**Tg6.0**
T	C	G	C	G	A	C	C	C	T	A	T	G	**Tg6.1**
T	T	G	C	G	A	C	C	C	T	G	T	G	**Tg6.2**
T	A	G	C	G	A	C	C	C	T	G	T	G	**Tg7.0**

**Table 2:** 12S rRNA haplotypes in captive and wild-caught *Testudo graeca*.

**12S haplotype**	**Number of individuals**	**Country of origin**	**Subspecies**
Tg1.0	33	Morocco (Al-Hoceima)/Tunisia/unknown/Algeria	*Testudo graeca graeca*
Tg1.1	1	Algeria	*Testudo graeca graeca*
Tg2.0	33	Libya/Tunisia/Italy (Sardinia)/unknown	*Testudo graeca graeca*
Tg2.1	1	Algeria	*Testudo graeca graeca*
Tg3.0	5	Unknown	*Testudo graeca graeca*
Tg3.1	1	Unknown	*Testudo graeca graeca*
Tg4.1	5	Unknown	*Testudo graeca whitei*
Tg4.2	8	Western Morocco (Laroche)/unknown	*Testudo graeca whitei*
Tg4.3	1	Unknown	*Testudo graeca whitei*
Tg5.0	17	West-Morocco (Laroche)/unknown	*Testudo graeca whitei*
Tg6.0	34	Lebanon/Israel/Turkey/Italy (Sardinia)/Bulgaria/unknown	*Testudo graeca terrestris*
Tg6.1	1	Unknown	*Testudo graeca terrestris?*
Tg6.2	1	Turkey	*Testudo graeca anamurensis?*
Tg7.0	17	Bulgaria/Russia/unknown	*Testudo graeca ibera*

**Table 3:** Nucleotide variation in a mt D-loop fragment of 22 Testudo graeca

**Tortoise no.**	**Nucleotide position**																				**Origin**	**12S haplotype**
					**1**	**1**	**1**	**1**	**2**	**2**	**2**	**2**	**2**	**2**	**3**	**3**	**3**	**3**	**3**	**3**		
	**4**	**4**	**5**	**9**	**1**	**7**	**7**	**9**	**0**	**0**	**3**	**4**	**5**	**9**	**1**	**1**	**2**	**3**	**7**	**7**		
	**8**	**9**	**8**	**9**	**3**	**0**	**4**	**3**	**1**	**8**	**6**	**4**	**8**	**4**	**3**	**6**	**2**	**2**	**5**	**7**		

TGKL	T	T	-	C	A	T	G	A	A	G	A	G	A	A	A	C	A	A	G	C	Morocco (Al-Hoceima)	Tg1.0
703888	T	T	-	C	A	T	G	A	A	G	A	G	A	A	A	C	A	A	G	C	Tunisia	Tg1.0
704089	T	T	-	C	A	T	G	A	A	G	A	G	A	A	A	C	A	A	G	C	Morocco	Tg1.0
SNA1	T	T	-	C	A	T	G	A	A	G	A	G	G	A	A	C	A	A	G	C	Algeria	Tg1.0
TGG0042	A	C	-	T	A	T	G	A	A	G	G	G	A	A	A	C	A	A	G	C	Tunisia (Sibkur)	Tg2.0
TGG0043	A	C	-	T	A	T	G	A	A	G	G	G	A	A	A	C	A	A	G	C	Tunisia (Sibkur)	Tg2.0
TGG0041	A	C	-	T	A	T	G	A	A	G	G	G	A	A	A	C	A	A	G	C	Tunisia	Tg2.0
A2SN	A	C	-	T	A	T	G	A	A	G	G	G	A	A	A	C	A	G	G	C	Algeria	Tg2.0
TGGLY1	A	C	-	C	A	T	A	A	A	G	A	A	A	A	G	C	A	A	G	C	?	Tg3.0
TGLKX	A	C	-	C	A	T	A	A	A	G	A	A	A	A	G	C	A	A	G	C	?	Tg3.1
TGLM1	A	C	-	C	A	T	G	A	A	G	A	A	A	A	A	C	A	A	A	C	Morocco (Laroche)	Tg5.1
TGLM2	A	C	-	C	A	T	G	A	A	G	A	A	A	A	A	C	A	A	A	C	Morocco (Laroche)	Tg4.2
703157	A	C	-	C	A	T	G	A	A	G	A	A	A	A	A	C	A	A	A	C	?	Tg4.1
TGTURBO	A	C	-	C	A	T	G	A	A	G	A	A	A	A	A	C	A	A	A	C	?	Tg5.0
F1621	A	C	T	C	C	T	G	A	A	G	G	A	A	A	A	T	A	A	A	C	Lebanon	Tg6.0
HZTGIAN	A	C	-	C	C	T	G	A	A	G	G	A	A	G	A	T	A	A	A	C	South-Turkey	Tg6.0
TGRW5	A	C	-	C	C	T	G	A	A	G	G	A	A	G	A	T	A	A	G	T	Israel (Hasharon Park)	Tg6.0
TGRW4	A	C	-	C	C	T	G	A	A	G	G	A	A	G	A	T	A	A	A	T	Israel (Golan Heights)	Tg6.0
TGRW1	A	C	-	C	C	T	G	A	A	G	G	A	A	G	A	T	A	A	A	T	Israel (Petah Tiqwa)	Tg6.0
TGRW3	A	C	-	C	C	T	G	A	T	G	G	A	A	G	A	T	A	A	A	T	Israel (Golan Heights)	Tg6.0
TGRW2	A	C	-	C	C	T	G	A	A	A	G	A	A	A	A	T	A	A	G	T	Israel (Petah Tiqwa)	Tg6.0
703837	A	C	-	C	C	C	G	G	A	A	G	G	A	A	A	T	G	A	G	C	?	Tg7.0

### Phylogenetic reconstitutions

**Figure 1:**
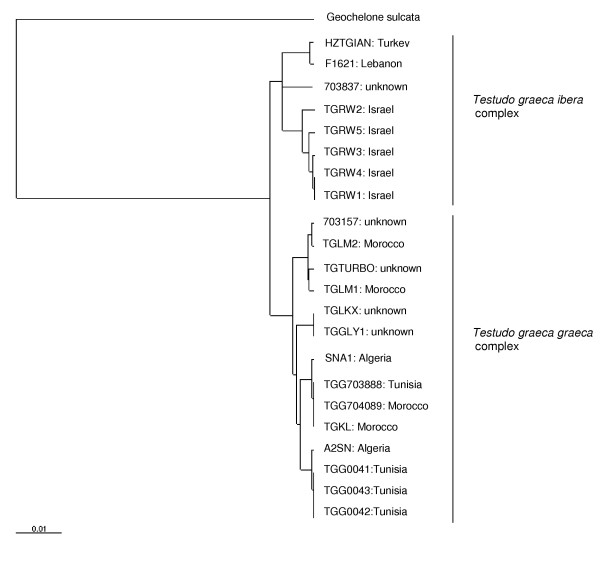
**ML tree of combined 12S rRNA (394 nt) and D-loop (411 nt) sequences from *Testudo graeca*. **Corresponding sequences from *Geochelone sulcata *were used as outgroup. Provenance of tortoise samples is indicated. The Ln likelihood of the tree = -1708.32.

**Figure 2:**
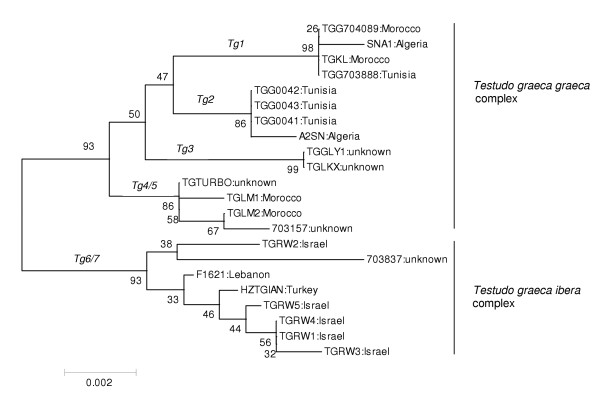
**PNJ tree of combined 12S rRNA (394 nt) and D-loop (411 nt) sequences from *Testudo graeca*. **Provenance of the samples is indicated. Five hundred bootstrap replicates have been analysed. Numbers shown are bootstrap confidence levels (BCL). Haplotype designations are indicated.

The 12S rRNA gene data set consisted of 394 total characters of which 377 were constant and 8 were parsimony informative. The D-loop data set consisted of 411 total characters of which 391 were constant and 13 were parsimony informative. Both data sets were analysed separately, as well as combined, where the 3'end of the 12S sequence was joined to the 5'end of the D-loop sequence (= 805 nt of total sequence). Analyses of both fragments resulted in a comparable clustering of haplotypes (not shown). Only the analysis of the combined data set will be discussed here. Two main clusters, present in both the ML (Fig. 1) and NJ (Fig. 2) trees, each supported by bootstrap values of 93 in the NJ tree, consisted of *T. graeca *from Morocco, Tunisia and Algeria (subspecies *T. g. graeca *and *T. g. whitei*), and *T. graeca *from Israel, Lebanon, and Turkey (*T. g. terrestris *and *T. g. ibera*), respectively. Four *T. g. graeca *subclades are detected with both ML and NJ methods, all four receive high support (bootstrap value ≥ 85) with the NJ method. These lineages could represent proposed subspecies, but unfortunately detailed descriptions or geographic origins of the animals sequenced were frequently lacking. The most divergent subclade (comprising numbers 703157, TGLM2, TGTURBO, and TGLM1) corresponds with the haplotype from *T. g. whitei *[8] and to the West Moroccan haplotype of Álvarez *et al. *[10]. Within *T. g. terrestris / ibera *no subclades could be confidently resolved. One wild-caught animal described as *T. graeca nikolskii *did harbour 12S haplotype Tg7.0, which is common in *T. g. ibera*. In three animals described as putative *T. graeca anamurensis*, three different 12S haplotypes were found: Tg6.0, Tg7.0 and Tg6.2. Tg6.0 is the common haplotype of *T. g. terrestris*, while Tg7.0 is encountered in animals found north of Tg6.0, which are described as *T. g. ibera*. Most likely, two of these three animals are actually *terrestris *or *ibera*, also because their geographic origins are unknown. Since *T. graeca anamurensis *has a limited distribution in the south of Turkey, the unique haplotype Tg6.2 (differing by 2 nt from its nearest neighbour) could eventually belong to this putative subspecies. To confirm this finding, additional wild-caught animals should be analysed. It is possible that additional mt haplotypes are present in animals from the northeastern part of the range of *T. graeca *(e.g. the Balkans and Greece), but unfortunately no such samples were available.

### Nucleotide distances

P-distances were calculated to estimate the average amount of haplotype divergence within clades and between the five well-supported clades. The average distance for the whole data set was 0.010, with the average p-distance between the Tg6/7 haplotypes being 0.004. The four clades characterized by haplotypes Tg1, Tg2, Tg3, and Tg4/5 (*T. g. graeca*) showed less within-clade variation (0.000–0.002), with an overall average distance of 0.007. The average p-distance between haplotypes Tg6/7 and the other haplotypes was 0.014. So, the highest variation in this data set is found between haplotypes Tg6/7 versus all the other haplotypes, with a lower variation within the five supported clades.

### Dating of mitochondrial DNA lineages

*T. graeca *12S rRNA gene sequences from different locations grouped together when compared to 12S fragments from other tortoise species, suggestive of a single species [8]. At least 16 closely related but distinct mitochondrial lineages are found in *T. graeca *over the main part of its distribution range, suggestive of a relatively recent, rapid radiation of mitochondrial haplotypes in this species. Earlier, depending on the 12S rRNA substitution rate, we have calculated the divergence of *T. g. graeca *and *T. g. ibera *to occur between 0.3 and 2.3 million years ago [8]. Based upon fossil evidence, Mlynarski [14] dates the emergence of *T. cf. graeca *to the early Pleistocene, with the modern subspecies arising in the Holocene. The mitochondrial DNA data agree with a recent origin of *T. graeca *mt lineages (this paper and [8]). Although the exact dating of lineage separation depends upon the (unknown) substitution rate of tortoise mitochondrial DNA, the foundation of the modern lineages is most likely in the Pleistocene (between 0.1 and 1.8 mya). Especially the very clear separation of *T. g. graeca *and *T. g. ibera *is compatible with an earlier origin of these subspecies than the Holocene.

MtDNA variation in *T. graeca *is rather large compared with the related species *T. hermanni*, probably because no losses occurred due to Pleistocene Ice Age selection as a consequence of the geographic distribution of *T. graeca *compared with that of the European *T. hermanni *[8]. This variety in mitochondrial lineages in both *T. g. graeca *and *T. g. ibera*, although not representing subspecies per se, could be at the bottom of the confusing taxonomy of *T. graeca*. The mtDNA variation observed corresponds with ongoing evolution, and is most likely accompanied by nuclear gene evolution or allele selection that could result in subtle morphological changes, especially when there is simultaneous pressure by environmental conditions.

It is interesting to note that *T. graeca *12S fragment is somewhat less variable than the D-loop fragment analysed, a finding compatible with the situation in mammals, where rRNA genes evolve slower than synonymous sites and the variable parts of the D-loop [15]. Also, strong rate variability among mammalian species was detected for the D-loop region [15], making this region less suitable for interspecies comparisons. The average amount of nucleotide substitutions between the *T. g. graeca *and *T. g. ibera *haplotype sets is 0.011 subst/site for the 12S gene, and 0.017 subst/site for the D-loop fragment.

In Testudines, the overall mtDNA sequence divergence rate has been estimated at 0.4–0.6% per million years [16,17]. Others, however, did not find this 2.5–3 × reduced rate for the 12S rRNA gene in turtles [18] and other reptiles [19]. Palkovacs *et al. *[20] detected an accelerated rate in Malagasy *Pyxis*, which they attributed to small body size and a short generation time in this species. In contrast, mammalian body size, generation time, and metabolic rate were not found to influence "taxon specific" mtDNA evolutionary rates [15]. If we assume that the divergence of *T. g. graeca *and *T. g. ibera *occurred approximately in the middle of the Pleistocene (± 1 million years ago), the nucleotide substitution rate in *T. graeca *is estimated to be around 1.1% per million years for the 12S gene, and 1.7 % per million years for the D-loop fragment (= mammalian rate). If we assume the time point of divergence to be earlier (e.g. 1.8 mya, the beginning of the Pleistocene), substitution rates decrease to 0.6% per million years for the 12S gene, and 0.9 % per million years for the D-loop fragment (=decreased reptile rate).

Some authors (e.g. [11]) have proposed to elevate *T. g. graeca *and *T. g. ibera *to full species status (*T. graeca *and *T. ibera*). Full species status is compatible with our mtDNA data, and would certainly simplify *T. graeca *taxonomy. Full species status would also be compatible with geography, *T. graeca *being confined to North Africa, and *T. ibera *to the remaining regions in Europe, the Middle East and countries belonging to the former Soviet Union, with Egypt serving as a corridor where neither *T. graeca *nor *T. ibera *is found. However, as the evolutionary history of a species can be confused by hybridisation, introgression, and incomplete lineage sorting [21], analysis of nuclear markers would be advisable before full-species status can be reached.

## Conclusion

Based upon fragments of the mitochondrial 12S rRNA gene and a D-loop fragment, 16 different haplotypes were detected in animals of the tortoise species *Testudo graeca *obtained from several locations in North Africa, the Middle East and Turkey. Phylogenetic analysis suggested a clear separation between the recognized subspecies *T. g. graeca *and *T. g. ibera*, which a calculated divergence time in the early or middle Pleistocene. Many other putative subspecies were not clearly recognizable from their mt haplotypes, and clustered either with *T. g. graeca *or *T. g. ibera *haplotypes. Awaiting analysis of nuclear markers, we propose full species status for *T. g. graeca *(*T. graeca*) and *T. g. ibera *(*T. ibera*), based upon their divergent mtDNA lineages, with subspecies to be defined.

## Methods

### Tortoise samples

A total of 158 *Testudo graeca *samples from supposedly unrelated individuals (verified only for captive animals)were obtained from Henk Zwartepoorte (Blijdorp Rotterdam Zoo, Rotterdam, The Netherlands), Donato Ballasina (CARAPAX, Massa Marittima, Italy), Richard Gibson (Durrell Wildlife Conservation Trust, Jersey, UK), Ron Winkler (University of Leiden, Leiden, The Netherlands), Adrie van Zanten (Dierenpark Amersfoort, Amersfoort, The Netherlands), and from several individual members of the Nederlandse Schildpadden Vereniging (Waalwijk, The Netherlands). Samples were obtained from both captive animals of unknown provenance as well as wild-caught specimens from known geographic origin (Table 1).

### Amplification and sequencing

Tortoise mucosal cell samples were obtained by gently scraping the inside of the mouth with a cotton swab or plastic scraper, a non-invasive and highly efficient method for obtaining a sample for DNA analysis. In a few cases blood samples were taken. DNA was extracted by a procedure using silica and guanidine thiocyanate [22]. Amplification of 394 nucleotides of the mt 12SrRNA gene was done with the primer set 12S-L01091 / 12S-H01478 [23]; the numbering refers to the position in the human mtDNA. PCR primers were extended with T7 and SP6 promoter sequences, respectively, to facilitate direct sequencing of the PCR product. Amplification of 411 nucleotides of the mt D-loop sequence was done with a primer set consisting of upstream primer myt001 (5' GAGAAAGACTTAAACCTTC 3'), and downstream primer myt003 (5' GACAAAACAACCAAAGGCCAG 3'), based upon corresponding sequences from *Geochelone nigra *(Genbank accession numbers AF192942 – AF192964). Primer myt001 is located in the mt tRNA^Pro ^gene, primer myt003 in the D-loop sequence. PCR primers myt001 and myt003 were extended with -21M13 and M13RP sequences, respectively, to facilitate direct sequencing of the PCR product.

PCR amplifications were done using the following protocol: denaturation 5 min 95°C, amplification 35 cycles of 1 min 95°C, 1 min 55°C, 2 min 72°C, followed by an extension of 10 min 72°C. Sometimes 40 cycles were needed to obtain a D-loop fragment.

Sequencing was performed in both directions using a PE – Applied Biosystems 377 automated sequencer, using the Dyenamic Direct Cycle Sequencing Kit and the Dyenamic Energy Transfer Dye Primer Set from Amersham Int. (UK), following the manufacturers protocols. Mitochondrial D-loop and 12S fragment sequences used in the analyses are available as supplementary information (see additional file 1).

### Phylogenetic analysis

Obtained sequences were aligned manually. Neighbor joining (NJ) trees of the sequences and reference sequences were constructed using the Kimura 2-parameter method and the NJ option in the MEGA package [24]. Bootstrap confidence levels were calculated for 500 replicates of the NJ tree. A single nucleotide insertion in a single D-loop sequence was treated as additional information, and used in pair-wise comparison. Furthermore, the data were analysed using Felsenstein's maximum-likelihood (ML) method as implemented in the BioEdit program, version 4.7.8 (kindly provided by Tom Hall, Department of Microbiology, North Carolina State University, 1999). The option DNAML version 3.5c was used with the transition / transversion ratio set at 2.0. Due to computational time, no bootstrap levels were calculated for the ML tree. P-distances were calculated with the distance option of the MEGA program.

## Authors' contributions

ACvdK conceived and designed the study, analysed the sequences, and drafted the manuscript. DLPhB initiated the studies on DNA variation in tortoise species. FZ carried out the PCR reactions, and performed the sequence analysis.

## Supplementary Material

Additional File 1 *Testudo graeca *mitochondrial nucleotide alignments Nucleotide sequences of *Testudo graeca *mitochondrial D-loop and 12S fragments Click here for file
